# Is Chinese Spring Festival a key point for glycemic control of patients with type 2 diabetes mellitus in China?

**DOI:** 10.3389/fpubh.2022.975544

**Published:** 2022-12-22

**Authors:** Huilin Xu, Li Cao, Jun Li, Fen Zhang, Weijie Wang, Tongtong Liang, Xiaohua Liu, Chaowei Fu

**Affiliations:** ^1^Shanghai Minhang Center for Disease Control and Prevention, Shanghai, China; ^2^School of Public Health, Fudan University, Shanghai, China; ^3^NHC Key Laboratory of Health Technology Assessment, Fudan University, Shanghai, China; ^4^Key Laboratory of Public Health Safety, Fudan University, Shanghai, China

**Keywords:** type 2 diabetes mellitus, fasting blood glucose, glycemic control, Chinese Spring Festival, holiday effects

## Abstract

**Objectives:**

This study aims to explore the long-term trend of fasting blood glucose (FBG) among urban patients with type 2 diabetes mellitus (T2DM) and the impacts of the Chinese Spring Festival on their glycemic control in urban China.

**Methods:**

The general information and longitudinal monitoring data of patients with T2DM in Minhang District, Shanghai China from 15 December 2006 to 31 December 2015 were collected. The FBG records were grouped into three periods, namely, the preholiday period (2 months right before the Chinese Spring Festival), the holiday period (from 28 December to 7 January of the lunar calendar year), and the postholiday period (2 months after the Chinese Spring Festival). The Mann-Kendall trend test and Cochran-Armitage trend test were occupied to explore the long-term trend, and paired *t*-test and chi-square (χ^2^) test were used to determine the differences in glycemic level and control rate between the preholiday and postholiday periods, respectively.

**Results:**

From 2007 to 2015, the glycemic control rate in patients with T2DM showed an upward trend (*P* < 0.001), and the FBG level showed a decreasing trend (*P* = 0.048). After the Chinese Spring Festival, the glycemic control rate decreased significantly (*P* < 0.001), and the FBG level increased significantly (*P* < 0.001) compared to those during the preholiday period. The incidence of hypoglycemia increased during holidays. Patients who were aged 60–69 years, overweight or obese, with hypertension, with a disease duration of <3 years, or with poor glycemic control in one previous year were more likely to be affected by the holiday.

**Conclusion:**

Chinese Spring Festival is a key point for glycemic control of patients with T2DM in China. Intensive holiday-specific diabetic healthcare needs to be further improved, and community-based interventions should be developed and implemented to control the possible holiday effects.

## 1. Introduction

Diabetes is one of the leading causes of death worldwide and affected more than 536 million people in 2021 (1), while type 2 diabetes mellitus (T2DM) accounts for more than 90% (2). China had the most people with diabetes (140.9 million), and 1,396,662 deaths were attributed to diabetes in 2021 (3). Moreover, diabetes increases the risk of physical disability and life-threatening complications, of which coronary heart disease, cerebrovascular disease, and peripheral vascular disease are major causes of morbidity and mortality (4). The main risk factor for diabetic complications is poor glycemic control including hyperglycemia and glycemic fluctuation. Higher-than-optimal blood glucose causes even more deaths than diabetes, by increasing the risk of cardiovascular and other diseases (5, 6). Given the high prevalence of diabetes and the severity of poor glycemic control, both determining the most common risk factor and identifying individuals at risk of poor glycemic control are vital for the prevention of diabetic adverse events.

For most patients with T2DM, a proper dietary pattern, physical activity, and medication may be required to lower blood glucose to avert chronic complications. The majority of patients can be expected to aim for a hemoglobin A1c (HbA1c) value of 7.0% (53 mmol/mol), or alternatively, an fasting blood glucose (FBG) value of ≤ 7.0 mmol/L (126 mg/dl) (4). Despite good-quality evidence on the benefit of glycemic control, many patients could not reach recommended targets for glycemic levels. In China, only 24.8–39.7% of patients who received treatment for diabetes achieved HbA1c < 7.0% (7–9). There were many factors associated with glycemic control, such as age, self-management of stress, family and social supports (10), dietary patterns, physical activity, taking medications (11), smoking (12), and lipid profiles (13). Patients aged between 60 and 69 years, were obese, were active smokers, were lacking physical activity, or with more than one oral hypoglycemic agent were less likely to achieve the glycemic control target (14). Continuous glucose monitoring (15), monotherapy and good adherence (12), and health education (16) were found to have positive impacts on the achievement of good glycemic control.

The New Year holiday is an important period for glycemic control in western research (17), but the effects of the New Year holiday on glycemic control were still lacking among T2DM cases in China so far (18). During the holidays in China, patients are customarily physically inactive and enjoy salty meals and alcoholic beverages, which may result in hyperglycemia and glycemic fluctuation. However, although the issue of holiday effects on glycemic control seems to be of clinical importance, it has not been addressed by clinical practice guidelines, suggesting that further relevant evidence is needed. To date, we found only one study that had examined glycemic change over the Chinese Spring Festival among adults with T2DM in Taipei and found a significant increase of HbA1c over the period, demonstrating an adverse influence of holidays on glycemic control, but the effects of the New Year holiday on glycemic control seemed inconsistent among studies in China and abroad (18–21). A study conducted in Greece found an apparent peak in fasting glucose levels after Christmas and Easter months (17), which was similar to the studies carried out in Singapore (20) and England (22). However, no increase was observed in the mean FBG concentration over the Christmas period in the study of Rees et al. (19). Moreover, despite a large number of patients with diabetes in China, limited information is available on the status of glycemic control and management of this population. Therefore, it is necessary to assess the glycemic control status of urban Chinese patients with T2DM under the Diabetes Standardized Management Program (DSMP) (23) and understand the possible effects of the Chinese Spring Festival on glycemic control. Minhang District of < city>Shanghai < /city>, China covered 1,000,000 urban residents; of which, more than 50,000 of them with T2DM were enrolled in DSMP since 2004 (24). We aimed to describe the long-term trend of glycemic control status over 9 years from 2007 to 2015 and explore the possible effects of the Chinese Spring Festival on the glycemic control in this study population of urban Chinese patients with T2DM under DSMP.

## 2. Research design and methods

### 2.1. Study participants

The Diabetes Standardized Management Program was a Basic Public Health Service (BPHS) implemented in 2004 in the Minhang District of Shanghai under the nationwide Chinese Guidelines for Diabetes Prevention and Management (24). Subjects with T2DM diagnosed by the 1999 T2DM diagnostic criteria of the World Health Organization (25) were enrolled in this program and managed by the community health service centers in Minhang District from 2004 to 2015. Two grouping rules based on the Goals for Glycemic Control in patients with T2DM were designed for the standardized management procedure. Group 1 was followed up by general practitioners (GPs) monthly and was considered to have poor glycemic control, which means patients have an ideal (preprandial blood glucose: 4.4–6.1 mmol/L) or general (preprandial blood glucose: ≤ 7.2 mmol/L) level of glycemic control for less than three-quarters of the time during 1 year. Group 2 was followed up every 3 months and was defined as the good glycemic control group, in which patients have an ideal or general level of glycemic control for more than three-quarters of the time during 1 year. Group assignment for the first management year depended on the individual's first FBG values, with poor glycemic control (preprandial blood glucose: >7.2 mmol/L) was grouped into group 1, otherwise grouped into group 2. In the subsequent year, group assignment was assessed annually based on the individual's glycemic control level during the previous year. The study was approved by the Institutional Review Board of the Center for Disease Control and Prevention in Minhang District, Shanghai (No: EC-2021-014). Informed consent from participants involved in the study was waived because anonymized data compiled from electronic medical records were applied in the study.

To ensure each calendar year has a sufficient sample size and enough records for the analysis of the long-term trend of FBG among patients with T2DM in the Minhang District, study participants were restricted to those who were managed between 15 December 2006 and 31 December 2015. Records from 2007 to 2015 were used for the long-term trend analysis, and the additional records from 2006 were retained to explore the holiday effects of the Chinese Spring Festival. In this study, the Chinese Spring Festival holiday was about 10 days from 28 December to 7 January of the lunar calendar year as the holiday period (0 month). The preholiday period was defined as the 2 months before the holiday period (−2 to −1 month), the postholiday period was defined as the 2 months after the holiday period (1–2 months), and the recovery period was defined as the 2 months after the postholiday period (3–4 months). If there was more than one record within an analyzed month or period in a given year, the one closest to the holiday period was employed. The holiday influence period was defined as the duration from the beginning of the preholiday period to the end of the postholiday period (−2 to 2 months), and the rest of the year was defined as the holiday non-influence period. Participants included in the analysis of the Chinese Spring Festival effects were those who had at least one FBG record during the holiday influence period of any year from 2007 to 2015.

**Figure 1:**
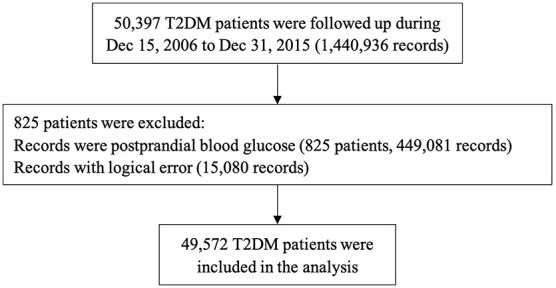
Flowchart for recruitment of study participants.

From 15 December 2006 to 31 December 2015, a total of 50,397 patients with T2DM were registered and managed by GPs. We further excluded 825 patients who collected postprandial blood glucose records only during the study period. Finally, 49,572 patients (23,276 men and 26,296 women) were involved in the data analysis of this study (Figure 1).

### 2.2. Baseline information and follow-up measurement of FBG

Baseline information, including demographics (sex, date of birth), daily physical activity, diabetic diet, self-reported standing height and body weight, date of diabetes diagnosis, comorbid hypertension, family history of T2DM, treatment pattern of antidiabetic drugs, and management group, was collected. Regular follow-ups of BPHS were carried out by GPs four or twelve times every year. All follow-up data were recorded in the form of electronic health records. A total of 976,775 follow-up records involving FBG values ranging from 0.6 to 33.3 mmol/L were selected for this study.

### 2.3. Definition

The body mass index was calculated as weight in kg divided by height in m squared and was divided into four groups: underweight (< 18.5 kg/m^2^), normal weight (18.5–23.9 kg/m^2^), overweight (24–27.9 kg/m^2^), and obesity (≥28 kg/m^2^) (26). The physical activity information was collected by the International Physical Activity Questionnaire (IPAQ), which is publicly available online (27). Hypertension was considered with systolic blood pressure (SBP) of ≥140 mmHg and/or diastolic blood pressure (DBP) of ≥90 mmHg, or self-reported hypertension or using anti-hypertensive medication (28). Diabetes mellitus (DM) was defined if any of the following conditions were met: fasting blood glucose of ≥7.0 mmol/L, 2-h post-meal blood glucose of ≥11.1 mmol/L, glycosylated hemoglobin of ≥6.5%, self-reported diabetes diagnosed by doctors, or receiving hyperglycemic treatment (29). Patients with FBG of ≤ 7.0 mmol/L were considered to reach the target for glycemic control (4).

### 2.4. Statistical analysis

Data were described as means and standard deviations (SDs) for continuous variables, and as the frequency with percentage for categorical variables. Baseline characteristics were summarized according to sex and compared between men and women using the χ^2^ test (for categorical variables) or *t*-test (for continuous variables). Paired *t*-test and χ^2^ test were used to determine the differences in glycemic level and control rate between the preholiday and postholiday periods, respectively. The Mann-Kendall trend test and Cochran-Armitage trend test were occupied to explore the long-term trend of FBG levels and glycemic control rates with calendar years from 2007 to 2015, respectively. Glycemic variation of the holiday influence period and non-influence period was evaluated using the fasting glucose SD and coefficient of variation (CV), and the SD ratio, CV ratio, and their 95% confidential intervals (CIs) of the holiday influence period and non-influence period were calculated. Stratified analyses were conducted to examine whether the potential holiday effect of glycemic control was moderated by the following variables: age, sex, BMI, physical activity, diabetic diet, duration of T2DM, treatment pattern, hypertension, and management group. The *p*-values for interaction were evaluated using the Breslow-Day test, and *p* < 0.2 was considered significant. Sensitivity analysis was performed with FBG records excluding those during the New Year's Day and/or the Tomb-Sweeping Day holiday. The holiday influence period for these two festivals was defined as from the 2 days before to 12 days after the festival (−2 to 12 days). All statistical procedures were performed using R version 4.0.3 (10 October 2020). All reported *p*-values were 2-sided, and *p* < 0.05 was considered significant except for the Breslow-Day test.

## 3. Results

### 3.1. Baseline characteristics of study participants

Among the 49,572 eligible patients with T2DM, the average age was 62.8 ± 10.6 years, ranging from 21 to 94 years, and more than half of the patients were women. The total follow-up duration for men and women was 81,711.4 and 97,501.0 person-years, respectively. Baseline information including sociodemographic factors, lifestyle behaviors, family history of T2DM, duration of T2DM, FBG at baseline, treatment pattern, comorbid hypertension, and group assignment is summarized in Table 1. There were significant differences in age, overweight or obese, diabetic diet, glycemic control, treatment pattern, hypertension, and management group between male and female patients at baseline.

**Table 1:** Baseline characteristics of participants.

**Characteristics**	**Total**	**Male**	**Female**	***t*/χ^2^ value**	**P-value**
	**(*n =* 49,572)**	**(*n =* 23,276)**	**(*n =* 26,296)**		
Age, years	62.8 ± 10.6	62.3 ± 10.8	63.3 ± 10.4	11.22	< 0.001
≤ 49	4,719 (9.5)	2,732 (11.7)	1,987 (7.6)	324.99	< 0.001
50–	14,157 (28.6)	6,243 (26.8)	7,914 (30.1)		
60–	17,327 (35.0)	8,316 (35.7)	9,011 (34.3)		
70–	10,160 (20.5)	4,655 (20.0)	5,505 (20.9)		
≥80	3,209 (6.5)	1,330 (5.7)	1,879 (7.1)		
BMI^*^, kg/m^2^	24.5 ± 3.1	24.5 ± 3.0	24.4 ± 3.3	1.03	0.304
< 18.5	1,047 (2.1)	429 (1.9)	618 (2.4)	99.88	< 0.001
18.5–	27,967 (57.3)	13,255 (57.8)	14,712 (56.9)		
25.0–	17,163 (35.2)	8,248 (36.0)	8,915 (34.5)		
≥30.0	2,522 (5.2)	968 (4.2)	1,554 (6.0)		
Physical activity^*^ (Yes)	30,124 (60.8)	14,141 (60.8)	15,983 (60.8)	0.53	0.467
Diabetic diet^*^ (Yes)	39,378 (79.4)	18,356 (78.9)	21,022 (79.9)	8.87	0.003
Family history of T2DM^*^ (Yes)	11,542 (23.3)	5,400 (23.2)	6,142 (23.4)	0.90	0.344
Duration of T2DM, years	1.9 ± 2.1	1.9 ± 2.1	1.8 ± 2.1	2.29	0.022
< 3.0	34,609 (69.8)	16,191 (69.6)	18,418 (70.0)	3.59	0.166
3.0–	11,890 (24.0)	5,593 (24.0)	6,297 (23.9)		
≥6.0	3,073 (6.2)	1,492 (6.4)	1,581 (6.0)		
FBG at baseline, mmol/L	7.8 ± 2.4	8.0 ± 2.5	7.7 ± 2.3	11.27	< 0.001
≤ 7.0	23,661 (47.7)	10,718 (46.0)	12,943 (49.2)	49.70	< 0.001
>7.0	25,911 (52.3)	12,558 (54.0)	13,353 (50.8)		
Treatment pattern^*^^#^				26.03	< 0.001
Insulin injection	2,958 (6.0)	1,578 (6.8)	1,380 (5.2)		
Sulphonylurea	19,963 (40.3)	9,800 (42.1)	10,163 (38.6)		
Biguanide	12,486 (25.2)	6,051 (26.0)	6,435 (24.5)		
Other oral drugs	6,071 (12.2)	2,917 (12.5)	3,154 (12.0)		
Hypertension (Yes)	35,439 (71.5)	16,168 (69.5)	19,271 (73.3)	88.34	< 0.001
Management group				75.71	< 0.001
Group 1	30,481 (61.5)	14,783 (63.5)	15,698 (59.7)		
Group 2	19,091 (38.5)	8,493 (36.5)	10,598 (40.3)		

BMI, body mass index; T2DM, type 2 diabetes mellitus; FBG, fasting blood glucose.

^*^Missing value.

^#^Drug combinations were adopted by some patients.

Data are presented as n (%) or mean ± SD.

### 3.2. Annual glycemic changes of community-managed patients with T2DM

**Figure 2:**
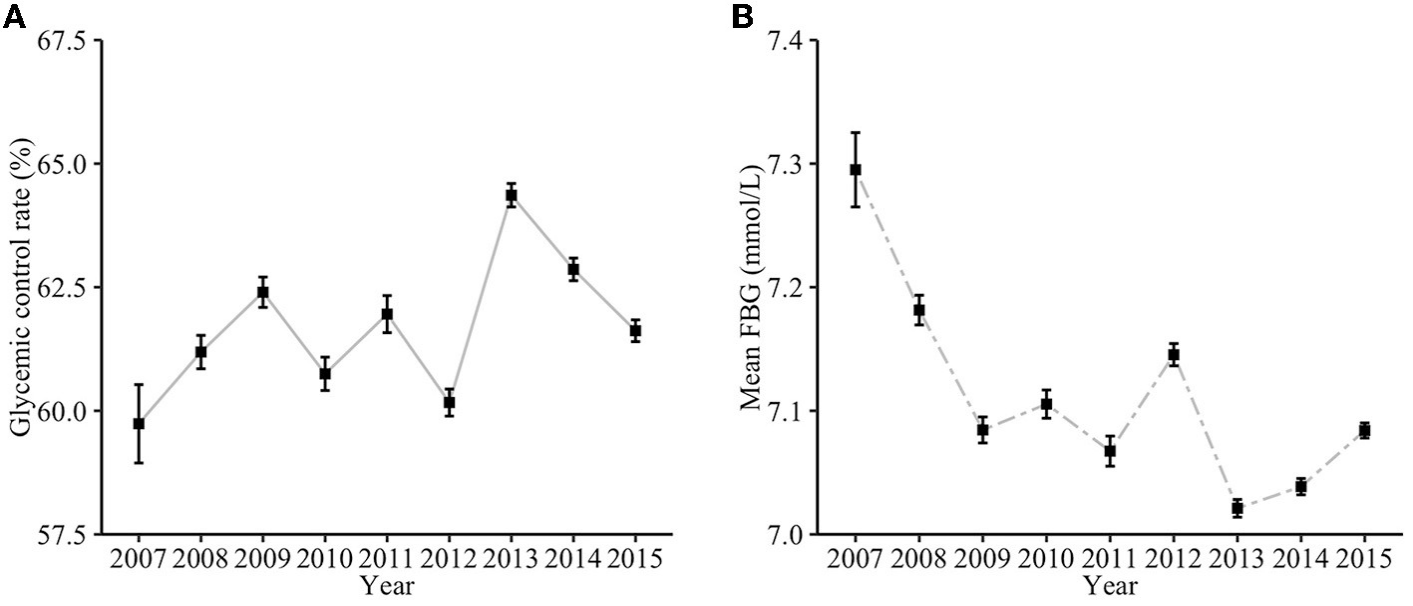
The annual glycemic trend of community-managed patients with T2DM from 2007 to 2015. **(A)** Glycemic control rate; **(B)** Mean FBG.

Between 2007 and 2015, the glycemic control rate varied from 59.7 to 64.4%, and the mean FBG ranged from 7.02 to 7.30 mmol/L. An increasing trend of glycemic control rate and a decreasing trend of mean FBG from 2007 to 2015 (Figure 2) were observed (*P* for trend: < 0.001 and 0.048, respectively). Patients with T2DM in the Minhang district had the best glycemic control rate of 64.4% in 2013 and the poorest glycemic control rate of 59.7% in 2007, and they were same measured by mean FBG value. From 2007 to 2015, the glycemic control rate increased by 1.9%, and the mean FBG level decreased by 0.2 mmol/L.

### 3.3. Effects of Chinese Spring Festival on glycemic control among community-managed patients with T2DM

**Figure 3:**
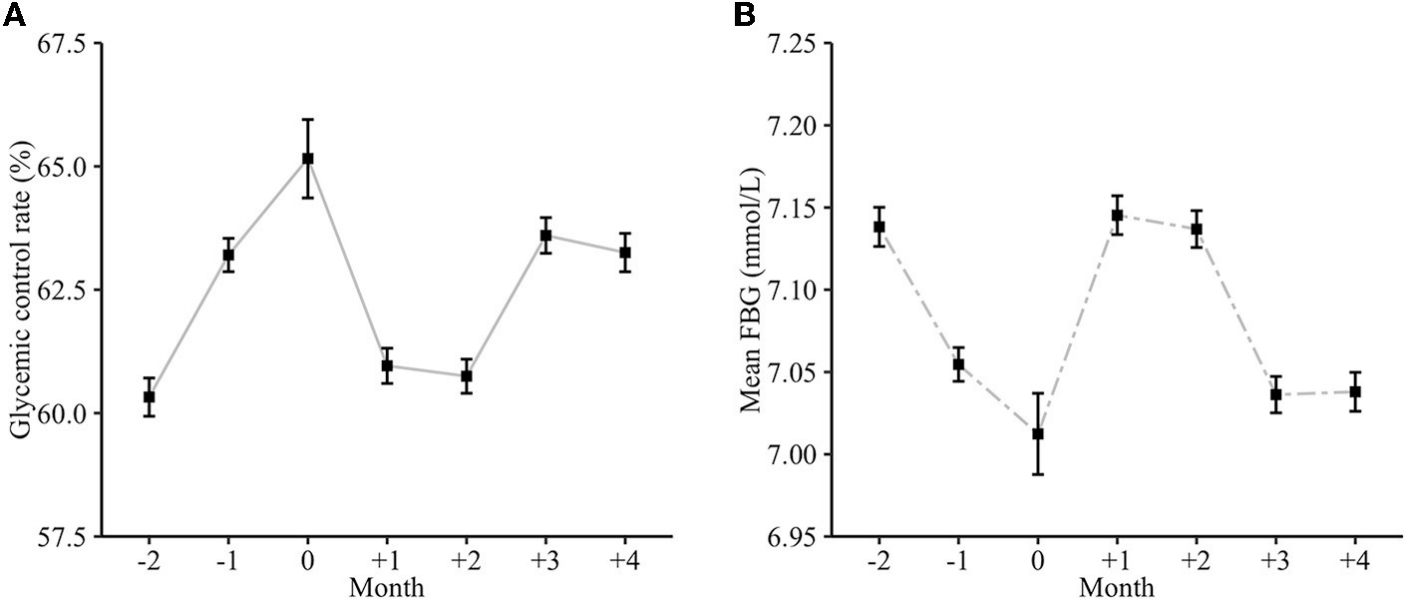
The monthly glycemic trend around the Chinese Spring Festival. **(A)** Glycemic control rate; **(B)** Mean FBG.

Blood glucose fluctuated for nearly 6 months around the Chinese Spring Festival (Figure 3) with the glycemic control rate from 60.3 to 65.2% and the mean FBG from 7.01 to 7.15 mmol/L. Such a rate increased during the preholiday period, reached a peak of 65.2% during the holiday, and then decreased until the third month after the holiday period. Correspondingly, the changes in mean FBG levels around the holiday were contrary to that. There were significant differences in the average glycemic control rate (65.0 vs. 63.1%, *P* < 0.001) and the mean FBG (6.92 mmol/L vs. 6.99 mmol/L, *P* < 0.001) between the preholiday period and postholiday period.

**Figure 4:**
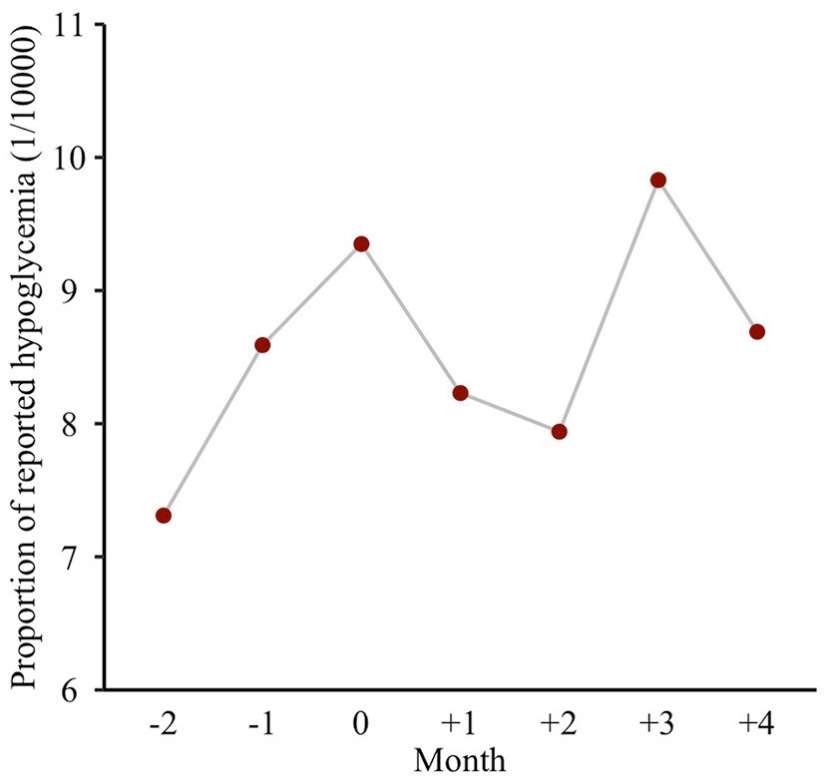
Monthly reported hypoglycemia around the Chinese Spring Festival.

The ratios (95%CIs) of SD and CV between the holiday influence period and non-influence period were 1.49 (1.46, 1.52) and 1.42 (1.40, 1.44), respectively, which indicated a significantly higher glycemic variation during the holiday influence period than the non-influence period. Also, the proportion of reported hypoglycemia ranged from 7.4/10,000 to 9.9/10,000 during nearly 6 months around the Chinese Spring Festival. As presented at Figure 4, the proportion during the holiday period was higher than that of the preholiday or postholiday periods.

### 3.4. Stratification analysis

Potential interactions between age group, sex, BMI, physical activity, diabetic diet, duration of T2DM, treatment pattern, hypertension, management group, and the Chinese Spring Festival effect on glycemic control were explored. The glycemic control rates among patients with T2DM significantly varied over BMI, duration of T2DM, and management groups, or were marginally different over age groups and hypertension, as shown in Table 2.

**Table 2:** Stratification analysis of the Chinese Spring Festival effects.

**Stratification variable**	**Period**	**Control**	**Not control**	**Glycemic control rate (%)**	**OR (95%CI)**	**P-value**
Total	Preholiday	70,751	38,025	65.0	1.00	–
	Postholiday	72,230	42,192	63.1	0.92 (0.90, 0.94)	< 0.001
**Age, years**						
≤ 49	Preholiday	4,358	3,169	57.9	1.00	–
	Postholiday	4,694	3,632	56.4	0.94 (0.88, 1.00)	0.055
50–	Preholiday	16,942	10,808	61.1	1.00	–
	Postholiday	17,742	12,203	59.3	0.93 (0.90, 0.96)	< 0.001
60–	Preholiday	26,067	12,991	66.7	1.00	–
	Postholiday	26,327	14,610	64.3	0.90 (0.87, 0.92)	< 0.001
70–	Preholiday	16,687	8,117	67.3	1.00	–
	Postholiday	16,557	8,650	65.7	0.93 (0.90, 0.97)	< 0.001
≥80	Preholiday	6,697	2,940	69.5	1.00	–
	Postholiday	6,910	3,097	69.1	0.98 (0.92, 1.04)	0.513
P for interaction						0.104
**BMI, kg/m** ^2^						
< 18.5	Preholiday	1,515	638	70.4	1.00	–
	Postholiday	1,560	674	69.8	0.97 (0.86, 1.11)	0.722
18.5–	Preholiday	40,251	20,375	66.4	1.00	–
	Postholiday	41,297	22,342	64.9	0.94 (0.91, 0.96)	< 0.001
25.0–	Preholiday	24,381	14,101	63.4	1.00	–
	Postholiday	24,758	15,932	60.8	0.90 (0.87, 0.92)	< 0.001
≥30.0	Preholiday	3,364	2,236	60.1	1.00	–
	Postholiday	3,371	2,504	57.4	0.89 (0.83, 0.96)	0.004
P for interaction						0.001
**Hypertension**						
Yes	Preholiday	54,188	28,843	65.3	1.00	–
	Postholiday	53,722	31,617	63.0	0.90 (0.89, 0.92)	< 0.001
No	Preholiday	16,563	9,182	64.3	1.00	–
	Postholiday	18,508	10,575	63.6	0.97 (0.94, 1.00)	0.092
P for interaction						0.120
**Duration of T2DM, years**						
< 3	Preholiday	24,629	11,850	67.5	1.00	–
	Postholiday	25,102	14,556	63.3	0.83 (0.81, 0.85)	< 0.001
3–	Preholiday	26,196	14,084	65.0	1.00	–
	Postholiday	27,623	15,380	64.2	0.97 (0.94, 0.99)	0.016
≥6	Preholiday	19,926	12,091	62.2	1.00	–
	Postholiday	19,505	12,256	61.4	0.97 (0.94, 1.00)	0.033
P for interaction						< 0.001
**Management group**						
Group 1	Preholiday	18,959	25,403	42.7	1.00	–
	Postholiday	17,856	29,952	37.3	0.80 (0.78, 0.82)	< 0.001
Group 2	Preholiday	51,792	12,622	80.4	1.00	–
	Postholiday	54,374	12,240	81.6	1.08 (1.05, 1.11)	< 0.001
P for interaction						< 0.001

OR, odds ratio; 95%CI, 95% confidence interval.

### 3.5. Sensitivity analysis

The findings were similar to the FBG records excluding those during New Year's Day (−2 to 12 days) and/or the Tomb-Sweeping Day holiday (−2 to 12 days). Changes in glycemic control rate and mean FBG had similar trends to the main analysis above (Supplementary Figure 1). There were significant differences in the average glycemic control rate (63.2 vs. 62.1%, *P* < 0.001) and the mean FBG (6.95 to 7.02 mmol/L, *P* < 0.001) between the preholiday period and postholiday period.

## 4. Discussion

This study indicated that the Chinese Spring Festival had negative impacts on the glycemic control of community-managed patients with T2DM. Such holiday effects included increased mean FBG values, decreased glycemic control rates, greater long-term glycemic variations, and increased risks of hypoglycemia mainly among the elderly. Also, the Chinese Spring Festival increased higher risk of failure to glycemic control for those aged between 60 and 69 years, with T2DM of < 3 years, with hypertension, having relatively poor glycemic control throughout the previous 1 year, and with a BMI of ≥25.0 kg/m^2^.

A few studies have linked the New Year holiday effects with glycemic control among patients with T2DM (17–20, 22). One finding from this study was that the Chinese Spring Festival had negative impacts on glycemic control with mean FBG and glycemic control rates among community-managed patients with T2DM. One previous study conducted in Taipei found that both FBG and HbA1c values of Chinese patients with T2DM increased from preholiday visits to postholiday visits (18), in alignment with our findings. Possible explanations were that patients ignored their doctor-recommended diet and exercise, stayed up late for entertainment and were overfatigue, ate too much high-calorie food, gained weight (17), irregularly took medications, failed to monitor their glycemia timely, and reduced clinics visits during the holiday (30). The glycemic control rate did not decline in the holiday period compared to that in the preholiday period among this study population, which may have a lag effect of poor glycemic control by celebration activities and inadequate self-management during the holiday (20), while it significantly decreased in the postholiday period. Also, over 2 months after the postholiday period, it was observed that the increased mean FBG fell at the preholiday levels or lower, which suggested that most of them were managed and treated more intensively after the Chinese Spring Festival. The holiday-specific diabetes education was proven to be effective in improving glycemic control during the winter holidays in the Chinese population (16). However, more attention should be paid to the holiday effects, and special interventions should be developed and implemented before the Chinese Spring Festival among Chinese patients with T2DM. Furthermore, interactions between age, hypertension, overweight/obesity, shorter duration of T2DM, management group 1, and the holiday effects were observed on glycemic control in this study. More healthcare management and health education should be targeted at those subpopulations to reduce holiday effects on glycemic control in the future.

Another finding from this study was that there was a statistically greater glycemic fluctuation during the holiday influence period than that during the rest of 1 year. The ratios (and their 95%CIs) of SD and CV were 1.49 (1.46, 1.52) and 1.42 (1.40, 1.44), respectively. Aligning with our finding, previous studies have presented higher glycemic fluctuation over the nearly 4-month period around the New Year holiday (17, 20, 31). Both holiday-specific activities and colder temperatures were considered as possible explanations (20, 32). Such a state of fluctuating hyperglycemia lasted for more than 4 months, which increased the risk of occurrence and development of microvascular complications (33). Furthermore, such glycemic fluctuation even contributed more to chronic complications of diabetes than hyperglycemia (34–36). So, intensive management should be taken around the Chinese Spring Festival to flatten the glycemic fluctuation and to improve glycemic control among Chinese patients with T2DM.

Also, the occurrence of hypoglycemia was reported among our study population, and it was found that patients were more likely to suffer from hypoglycemia during the holiday period, which was similar to one previous study (37). An excessive, prolonged, and, above all, unusual physical exertion due to overindulgence in festive foods may represent a relevant cause of holiday hypoglycemia (37). Too much medication may be another possible explanation. Previous studies found a huge increase in metformin consumption during the Spring Festival and the Tomb-Sweeping Day holiday and increased mean fasting plasma insulin concentration in patients with T2DM immediately after Christmas (19, 38). Of note, most of the hypoglycemic events occurred in elderly patients of this study. One previous research indicated that distinct hypoglycemia unawareness in the presence of pronounced hypoglycemia induced reaction time prolongation in elderly patients with T2DM, and they had a high risk of suffering from severe hypoglycemic episodes (39). However, this holiday hypoglycemia may have been underestimated in our study due to the limited size of FBG records during the holiday period. The risk of hypoglycemia should be taken into account with the choice of antihyperglycemic therapy and glycemic control target for elderly patients although intensive glycemic control is beneficial for appropriate patients in the long term (40).

This study has both public health and clinical implications. It indicated the negative impacts of the holiday celebration activities and inadequate self-management on glycemic control, which manifested mainly as long-term glycemic elevation and variation, and the poor glycemic control might not be reversed during the summer and autumn months (18). High-quality evidence has demonstrated that intensive glycemic control substantially reduced the risk of diabetes-related complications and mortality (41–43). To the best of our knowledge, this study was the first study to explore the effects of the Chinese Spring Festival on glycemic control among community patients with T2DM in the Chinese mainland. Main findings were robust in the sensitivity analysis. However, several limitations need to be considered in this study. First, FBG was occupied to reflect the glycemic control of patients with T2DM due to the lack of a better index of HbA1c mostly as community registry-based studies. Multiple glycemic indexes should be collected in future studies. Second, there is no uniform definition for the Chinese Spring Festival to measure the length of the effective period and the weight of effect, which depends on subjective judgment (30). Since the statutory holiday intervals of the Chinese Spring Festival varied over every calendar year, its effects may overlap that of other fixed holidays including New Year's Day and Tomb-Sweeping Day. In this study, the findings were similar to the FBG records excluding both holidays, and it indicated that both holidays may bias slightly. Third, seasonality effects were not controlled in this study, and future studies should consider constructing a better-designed model to clarify the holiday effect of glycemic control due to seasonality (20, 32). Fourth, due to the uncertainty of patients' schedules during the festival, there was no requirement for a fixed measurement time, which may bias the findings with the consideration of any difference between the first day and the last day of the festival, although blood glucose generally remains steady in some days. In addition, as an observational study, no causal relationship was explored in this study. Finally, due to the limitations of the observational study, the possible reasons for the negative holiday effects on glycemic control cannot be clarified in this study. Thus, we provided possible explanations based on previous studies and understandings of Chinese culture. Anyway, our findings called for studies with a better-designed model, diverse glycemic indexes, different populations, and high-level evidence to clarify whether and to what degree the holiday effects on glycemic control among different populations, and the reasons behind it.

## 5. Conclusion

Glycemia was not well controlled around the Chinese Spring Festival although there was good glycemic control overall among cases with T2DM in urban China from 2007 to 2015. The negative impacts of the Chinese Spring Festival on glycemic control mainly manifested as long-term glycemic elevation and variation, as well as increased possibility of hypoglycemia in the elderly. Therefore, intensive holiday-specific diabetic healthcare needs to be further improved, and community-based interventions should be developed and implemented to control the possible holiday effects.

## Data availability statement

The raw data supporting the conclusions of this article will be made available by the authors, without undue reservation.

## Ethics statement

The studies involving human participants were reviewed and approved by the Institutional Review Board of Center for Disease Control and Prevention in Minhang District, Shanghai (No: EC-2021-014). Written informed consent for participation was not required for this study in accordance with the national legislation and the institutional requirements.

## Author contributions

HX, XL, and CF conceived the idea and were responsible for the primary study design. JL, FZ, WW, and TL were involved in data acquisition and data verification. LC and HX contributed to the data analysis, the results interpretation, drafting, and revision of the manuscript. JL, FZ, WW, TL, XL, and CF contributed to the results interpretation and manuscript revision. All authors reviewed and agreed to the published version of the manuscript.
