# Supplementary material for: Is Chinese Spring Festival a key point for glycemic control of patients with type 2 diabetes mellitus in China?
Source: Front Public Health. 2022 Dec 22;10:975544. doi: 10.3389/fpubh.2022.975544 (PMC9813744; doi:10.3389/fpubh.2022.975544)
Supplement: Supplementary file 1 [file Data_Sheet_1.ZIP › Supplementary Material/Figure 3.pdf]

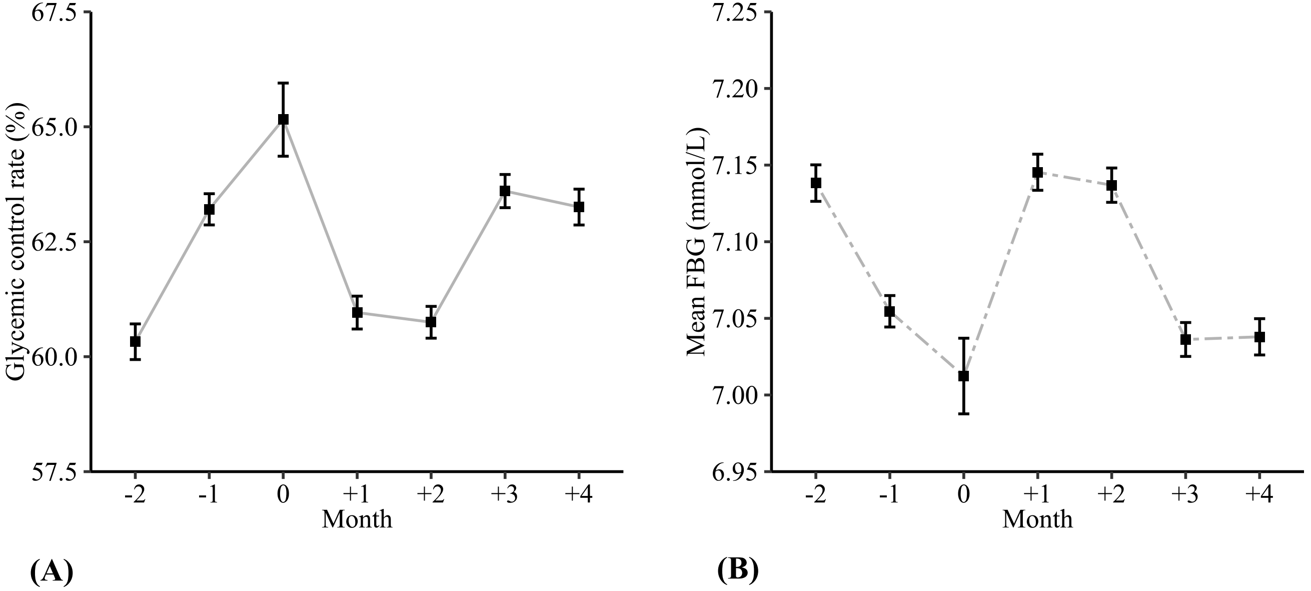

Figure 3. Monthly glycemic trend around the Chinese Spring Festival. (A) Glycemic control rate; (B) Mean FBG.
