# Supplementary material for: Is Chinese Spring Festival a key point for glycemic control of patients with type 2 diabetes mellitus in China?
Source: Front Public Health. 2022 Dec 22;10:975544. doi: 10.3389/fpubh.2022.975544 (PMC9813744; doi:10.3389/fpubh.2022.975544)
Supplement: Supplementary file 1 [file Data_Sheet_1.ZIP › Supplementary Material/Figure 2.pdf]

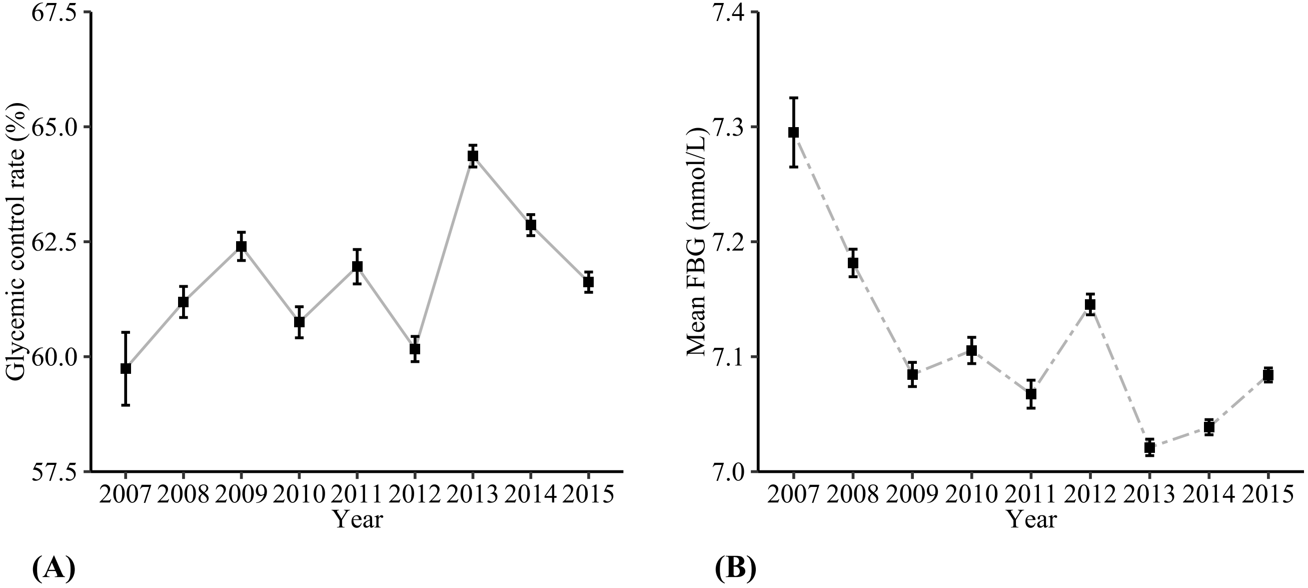

Figure 2. Annual glycemic trend of community-managed T2DM patients from 2007 to 2015. (A) Glycemic control rate; (B) Mean FBG.
