# Supplementary material for: Is Chinese Spring Festival a key point for glycemic control of patients with type 2 diabetes mellitus in China?
Source: Front Public Health. 2022 Dec 22;10:975544. doi: 10.3389/fpubh.2022.975544 (PMC9813744; doi:10.3389/fpubh.2022.975544)
Supplement: Supplementary file 1 [file Data_Sheet_1.ZIP › Supplementary Material/Supplementary Figure 1.docx]

Supplementary Material

# Supplementary Figures and Tables

For more information on Supplementary Material and for details on the different file types accepted, please see [here](http://home.frontiersin.org/about/author-guidelines#SupplementaryMaterial). Figures, tables, and images will be published under a Creative Commons CC-BY licence and permission must be obtained for use of copyrighted material from other sources (including re-published/adapted/modified/partial figures and images from the internet). It is the responsibility of the authors to acquire the licenses, to follow any citation instructions requested by third-party rights holders, and cover any supplementary charges.

## Supplementary Figures


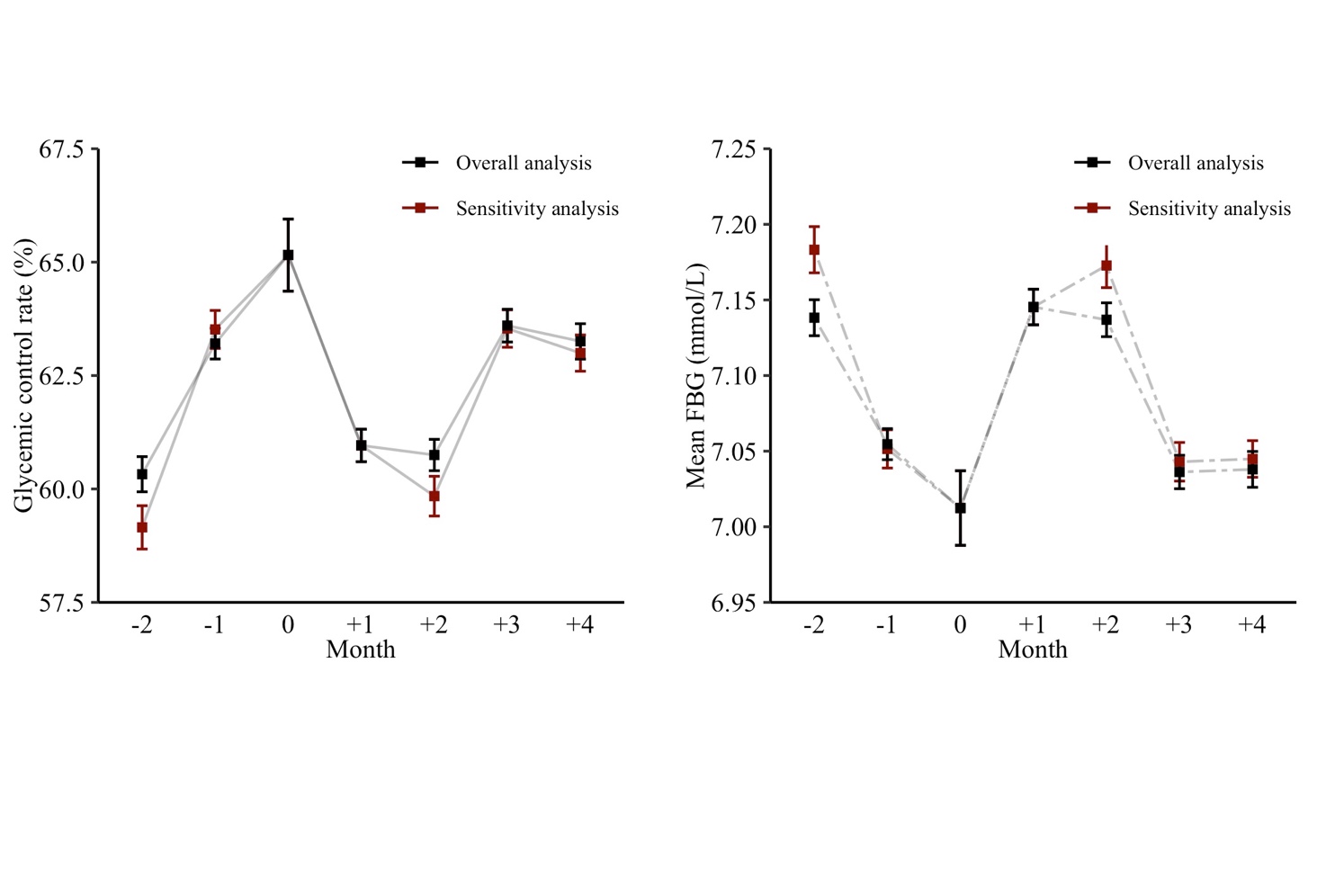


**(A) (B)**

**Supplementary Figure 1.** Sensitivity analysis when excluding FBG records affected by the New Year's Day and/or the Tomb-Sweeping Day holiday (132106 person-years remained). (**A**) Glycemic control rate; (**B**) Mean FBG.
