# Supplementary material for: Is Chinese Spring Festival a key point for glycemic control of patients with type 2 diabetes mellitus in China?
Source: Front Public Health. 2022 Dec 22;10:975544. doi: 10.3389/fpubh.2022.975544 (PMC9813744; doi:10.3389/fpubh.2022.975544)
Supplement: Supplementary file 1 [file Data_Sheet_1.ZIP › Supplementary Material/Supplementary Figure 1.pdf]

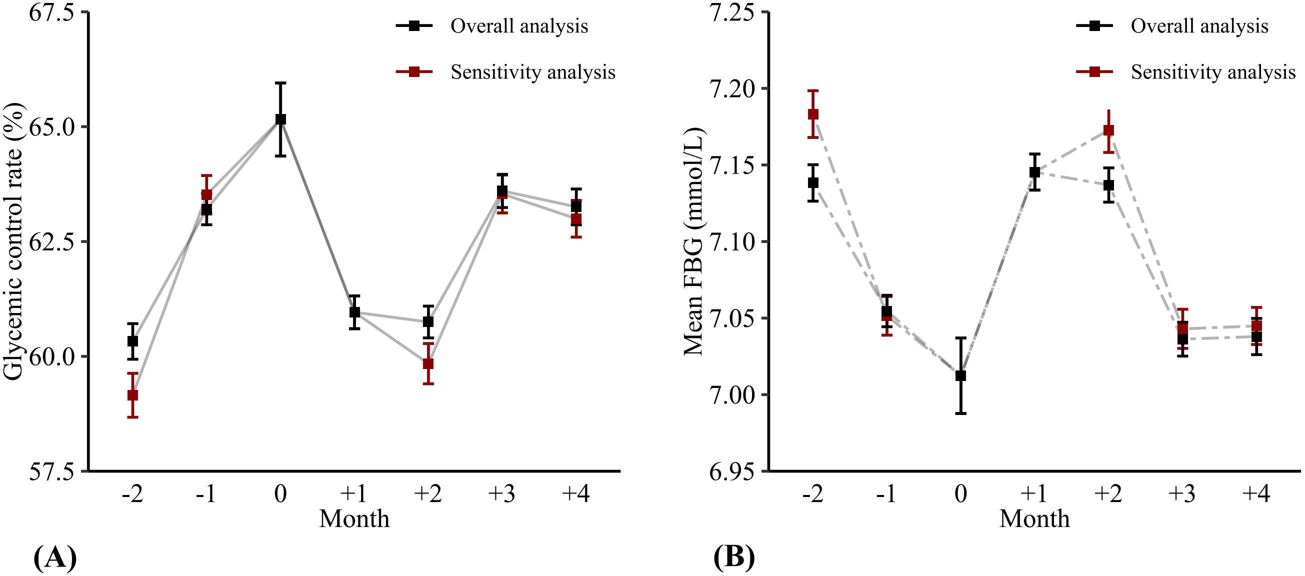

Supplementary Figure 1. Sensitivity analysis when excluding FBG records affected by the New Year's Day and/or the Tomb-Sweeping Day holiday (132106 person-years remained). (A) Glycemic control rate; (B) Mean FBG.
