# Supplementary material for: Is Chinese Spring Festival a key point for glycemic control of patients with type 2 diabetes mellitus in China?
Source: Front Public Health. 2022 Dec 22;10:975544. doi: 10.3389/fpubh.2022.975544 (PMC9813744; doi:10.3389/fpubh.2022.975544)
Supplement: Supplementary file 1 [file Data_Sheet_1.ZIP › Supplementary Material/Figure 1.docx]

825 patients were excluded:

Records were postprandial blood glucose (825 patients, 449,081 records)

Records with logical error (15,080 records)

50,397 T2DM patients were followed up during Dec 15, 2006 to Dec 31, 2015 (1,440,936 records)

49,572 T2DM patients were included in the analysis

**Figure 1**. Flowchart for recruitment of study participants.
